# Effect of COVID-19 pandemic on workflows and infection prevention strategies of endoscopy units in Hungary: a cross-sectional survey

**DOI:** 10.1186/s12876-021-01670-3

**Published:** 2021-03-03

**Authors:** Renáta Bor, Kata Judit Szántó, Anna Fábián, Klaudia Farkas, Mónika Szűcs, Mariann Rutka, Tibor Tóth, Anita Bálint, Ágnes Milassin, Zsolt Dubravcsik, Hungarian GI Endoscopy COVID-19 Study Group, Zoltán Szepes, Tamás Molnár

**Affiliations:** 1 First Department of Medicine, University of Szeged, Korányi Fasor 8-10, Szeged, 6720 Hungary; 2 Department of Medical Physics and Informatics, University of Szeged, Szeged, Hungary; 3 Department of Gastroenterology and Endoscopy, Bács-Kiskun County Hospital, Kecskemet, Hungary

**Keywords:** COVID-19, SARS-CoV-2, Pandemic, Personal protective equipment

## Abstract

**Background:**

Health care professionals in endoscopic labs have an elevated risk for COVID-19 infection, therefore, we aimed to determine the effect of current pandemic on the workflow and infection prevention and control strategies of endoscopy units in real-life setting.

**Methods:**

All members of Hungarian Society of Gastroenterology were invited between 7 and 17 April 2020 to participate in this cross-section survey study and to complete an online, anonymous questionnaire.

**Results:**

Total of 120 endoscopists from 83 institutes were enrolled of which 35.83% worked in regions with high cumulative incidence of COVID-19. Only 33.33% of them had undergone training about infection prevention in their workplace. 95.83% of endoscopists regularly used risk stratification of patients for infection prior endoscopy. While indications of examinations in low risk patients varied widely, in high-risk or positive patients endoscopy was limited to gastrointestinal bleeding (95.00%), removal of foreign body from esophagus (87.50%), management of obstructive jaundice (72.50%) and biliary pancreatitis (67.50%). Appropriate amount of personal protective equipment was available in 60.85% of endoscopy units. In high-risk or positive patients, surgical mask, filtering facepiece mask, protective eyewear and two pairs of gloves were applied in 30.83%, 76.67%, 90.00% and 87.50% of cases, respectively. Personal protective equipment fully complied with European guideline only in 67.50% of cases.

**Conclusions:**

Survey found large variability in indications of endoscopy and relative weak compliance to national and international practical recommendations in terms of protective equipment. This could be improved by adequate training about infection prevention.

## Background

Coronavirus disease 2019 (COVID-19) pandemic which declared by the World Health Organization on 11th March 2020 has a significant disruptive effect on the performance, workflow and safety of gastrointestinal endoscopy units in each affected country. COVID-19 is caused by a predominately respiratory pathogen, therefore the direct human to human transmission by respiratory droplets is one of the most important ways of the spread of infection [1, 2]. In addition, the detection of live virus in endoscopic biopsy specimens and stools is suggesting a possible fecal–oral transmission too [3, 4]. The surfaces of non-living objects could be contaminated by respiratory secretion and/or stool specimens where the virus could survive for hours to days ensuring the indirect transmission way of infection.

All endoscopic modalities should be considered aerosol-generating procedures because coughing and gagging usually occur during upper endoscopy and contact with liquid stool and passing flatus can happen in case of colonoscopy. Based on this, the infection risk of health care professionals in endoscopy units is outstandingly elevated. The general strategy for protection of both endoscopic staff and patients is the postponement of all non-essential endoscopic procedures, and only emergency endoscopies are permitted during pandemic [5–12]. However, national gastroenterological societies define urgent and non-postponable examinations somewhat differently depending on the local availability of human and material resources, national pandemic regulations and the phase of national pandemic alert. At the same time, these recommendations show great similarity in terms of proposed personal protective equipment. Considering the local regulations, in accordance with the European Society of Gastrointestinal Endoscopy (ESGE) guideline [5], Hungarian Society of Gastroenterology (HSG) recommended endoscopy for the management of acute, life-threatening diseases and for clinical conditions the elimination of which could potentially cause permanent health damage (Table 1). It also proposed the patients’ risk stratification prior to endoscopy and the minimization of the number of persons in the lab during examination, and determined the type of personal protective equipment (PPE). Furthermore, the local regulation strongly recommended the avoidance of at-risk healthcare professionals' direct contact with patients.

**Table 1:** Indication for endoscopic procedures and health professional personal protective equipment based on the recommendation Hungarian Society of Gastroenterology

Indications for endoscopic procedures during COVID-19 pandemic	
Acute life-threatening gastrointestinal disease	Severe cholangitis, acute biliary pancreatitis with cholangitis, biliary leakage
	Foreign body in upper gastrointestinal tract
	Acute gastrointestinal bleeding
Clinical conditions causing potentially permanent health damage if endoscopy is postponed	Suspicion of gastrointestinal malignancy (based on the results laboratory test, clinical status and/or cross-sectional image)
	Endoscopic intervention to ensure enteral feeding of patient if no other therapeutic option (malignant stricture stenting, percutaneous endoscopic gastrostomy)
	Endoscopic staging of cancers if the results is necessary for oncological or surgical treatment and not replaceable with other imaging modality
	Severe active ulcerative colitis

Health-professional personal protective equipment	
Low-risk patient	Surgical mask
	Gloves
	Disposable hairnet
	Protective eyewear
	Water-proof disposable gowns
High-risk or positive patient	FFP-2/3 mask
	Two pairs of gloves
	Disposable hairnet
	Protective eyewear
	Water-proof disposable gowns

*COVID-19* coronavirus disease 19, *FFP *filtering facepiece

The aim of this study was to assess the effect of COVID-19 pandemic on the workflow as well as infection prevention and control strategies of endoscopy units and to determine the adherence of Hungarian endoscopists to national and international recommendations in real-life settings based on the applied PPE and the medical indications of endoscopies.

## Methods

### Invitation of participants, study protocol

Questionnaire surveys-based multicentric nationwide study was carried out between 7 and 17 April 2020, more than six weeks after the beginning of COVID-19 epidemic in Hungary. Gastroenterologist members of HSG working in endoscopy units of primary, secondary and tertiary level medical centers were invited to complete an online questionnaire. The survey comprised of 40 questions which evaluated the effect of COVID-19 pandemic on the endoscopic laboratory workflows and assessed the infection control of endoscopic units (Additional file 1). We excluded partially completed and/or repeatedly submitted questionnaires, and those answers which were filled out by non-gastroenterologist members of HSG (Additional file 1).

### Ethical approval and consent to participate

Study protocol and the questionnaire which reflect our flexible approach during the COVID-19 pandemic were approved by the Scientific Research Ethics Committee of the Hungarian Medical Research Council (ETT TUKEB Registration No. IV/4669-2/2020/EKU). The invitation letter contained the aims of the survey, assured participants about the anonymity and strict confidentiality of data during the statistical analysis; it also emphasized that the participation is voluntary, and by completing the questionnaire, they contribute to the usage of obtained data for scientific purposes. The study was carried out under the Declaration of Helsinki.

### Primary aims of the study

The evaluation of the effect of COVID-19 pandemic on the operation of endoscopy units was the first major endpoint of the study of which we assessed the change of performance of individual endoscopists and the entire endoscopy unit (changes in the type and number of endoscopic procedures). On the other hand, we aimed to determine which endoscopy procedures should not be postponed according to the subjective opinion of Hungarian gastroenterologists, and these medical indications were compared with the recommendation of HSG and with the position statement of ESGE and European Society of Gastroenterology and Endoscopy Nurses and Associates (ESGENA). The local regulation strongly recommended the avoidance of at-risk healthcare professionals' direct contact with patients (e.g. over 65 years of age, severe respiratory or cardiovascular disease, chronic illness requiring immunosuppressant treatment, pregnancy), therefore, these people were ruled out from the gastroenterology care. We aimed to assess whether the lack of these people from work had any influence on the operation of endoscopy units.

We also evaluated the quality of infection prevention and control strategies of institutes. From this perspective, we wanted to assess both the (1) health care professionals related factors (e.g. training of endoscopy unit personnel on the prevention of COVID-19 infection, knowledge about the spread of infection and the correct use of PPE; adequate risk stratification of patients for potential COVID-19 infection), (2) disinfection protocols of endoscopy labs (negative-pressure rooms, adequate ventilation, cleaning the surfaces with virucidal agents, air purification) and (3) the usage of PPE (e.g. surgical mask, filtering facepiece [FFP]-2/3 mask, gloves, protective eyewear).

### Secondary aims of the study

The cumulative incidence of COVID-19 infection is showing large differences between counties in Hungary. Based on this, low- (< 20 infection per 100,000 inhabitants), medium- (20–40 infection per 100,000 inhabitants) and high-risk region (> 40 infection per 100,000 inhabitants) could be distinguished. Secondary endpoint was to compare the effect of COVID-19 pandemic on the endoscopy workflow and the quality of infection prevention and control strategies between institutes of regions with different cumulative COVID-19 incidence. In addition, we examined the changes in the above-mentioned factors between endoscopy units with different capacities. The capacity of labs was estimated based on the number of employed gastroenterologists. This way low- (less than 3 endoscopists), medium- (4 to 6 endoscopists) and large (more than 7 endoscopists) endoscopy units have been distinguished. Furthermore, we also examined the compliance of reported data about the workflows of endoscopy units with the recommendation of HSG and with the position statement of ESGE and ESGENA.

### Statistical analysis

Data for analysis were extracted from the online survey and recorded in Microsoft Excel. Statistical analysis was performed with Statistical Package for the Social Sciences software version 24 (SPSS Inc., Chicago, IL, USA). Descriptive statistics were performed on all studied variables which were expressed as means and medians with ranges. During the analysis of secondary endpoints, the differences between the workflows of endoscopy units were assessed by Fisher’s exact tests, a *p* value of < 0.05 was considered to indicate statistical significance.

## Results

All the 120 submitted questionnaires were filled out and no duplicates were found, therefore all of them were evaluated in the study. The obtained data represents the whole country because a total of 83 endoscopy units sent feedback, and at least one gastroenterologist in each of the 19 counties of Hungary completed the questionnaire. In each county, the number of participants in the survey closely correlated with number of endoscopy units. A total of 43 gastroenterologists work in endoscopy units of high-prevalence regions, while the rate of respondents from regions with medium and low risk of COVID-19 infection were 23.33% and 40.83%, respectively. The proportion of low-, medium and large-capacity endoscopy units were 39.17%, 28.33% and 31.67%, respectively. Total of 97.50% of the gastroenterologists claimed that they have read and are familiar with the recently published recommendation of HSG and the position statement of ESGE and ESGENA on gastrointestinal endoscopy and the COVID-19 pandemic.

### Effect of COVID-19 pandemic on the performance of endoscopy units

The operation of all endoscopic units was substantially affected by COVID-19 pandemic (Table 2). In 65.83% of endoscopy labs, at least one gastroenterologist was dropped out of work because their age or comorbidities would increase the risk of poor outcomes in case of acquisition of COVID-19 infection. Rate of employees who were dropped out of work was more than 20% in every third lab, however, the reduced staff number affected the endoscopy unit’s workflow only in 17.50% of cases. This can be explained by the fact that at the same time the number of examinations dramatically dropped due to a change in the medical indication of procedures. The number of endoscopies was halved in 90% of the labs; moreover, in 63.33% of the cases, the reduction exceeded 75%. Substantially lower proportion of labs reported decrease in number of endoscopic ultrasound (EUS; 67.65%) and endoscopic retrograde cholangiopancreatography (ERCP; 61.67%) examinations compared with other modalities (gastroscopy: 95.00%, colonoscopy: 91.67%); however, this difference might be caused by the fact that these techniques had not been available in every endoscopy unit.

**Table 2:** Results of survey regarding workflow and infection prevention and control strategies of endoscopic unit during COVID-19 pandemic

Results of cross-sectional survey (N = 120)	
Distribution of answers based on the regional cumulative incidence of COVID-19 infection	Low risk region: 43 (35.83%)
	Medium risk region: 28 (23.33%)
	High risk region: 49 (40.83%)
Distribution of answers based on the capacity of endoscopy unit	Low capacity lab: 47 (39.17%)
	Medium capacity lab: 34 (28.33%)
	High capacity lab: 38 (31.67%)
Decrease in the number of endoscopic examinations during COVID-19 pandemic	< 25% decrease: 2 (1.67%)
	25–50% decrease: 10 (8.35%)
	50–75% decrease: 32 (26.67%)
	> 75% decrease: 76 (63.33%)
Endoscopic modality the number of which is decreased during COVID-19 pandemic	Gastroscopy: 114 (95%)
	Colonoscopy: 110 (91.67%)
	ERCP: 81 (67.50%)
	EUS: 74 (61.67%)
Rate of endoscopists who were dropped out of work	< 20% of endoscopists: 73 (80.84%)
	20–40% of endoscopists: 22 (18.33%)
	> 40% of endoscopist: 15 (20.83%)
Rate of nurses who were dropped out of work	< 20% of nurses: 90 (75.00%)
	20–40% of nurses: 12 (10.00%)
	> 40% of nurses: 18 (15.00%)
Problems in relation with decreased number of healthcare professionals in endoscopy unit	It substantially affected the operation of endoscopy unit: 21 (17.50%)
	No negative effect was observed: 99 (82.50%)
Knowledge of Position Statement of ESGE and ESGENA	Yes: 117 (97.50%)
	No: 3 (2.50%)
Risk of endoscopic staff for COVID-19 infection	Increased: 118 (98.33%)
	Not increased: 2 (1.67%)
Which endoscopic modality has the highest risk for COVID-19 infection?	Risk of modalities is same: 54 (45.00%)
	Upper endoscopic examinations: 66 (55.00%)
	Lower endoscopic examinations: 0 (0.00%)
Training about prevention of COVID-19 infection in endoscopy unit	Yes: 40 (33.33%)
	No: 80 (66.67%)
Change in personal protective equipment in cases of high-risk patients	Mask: (41 (43.17%)
	Protective gown: 63 (52.50%)
	Nothing, because adequate personal protective equipment is not available: 14 (11.67%)
	Nothing, I am not afraid of infection: 2 (1.67%)
Minimization of number of persons in the lab during examination	Yes: 113 (94.17%)
	Administrator stays in the lab: 5 (1.67%)
	Young doctor stays in the lab, who is learning endoscopy: 2 (1.67%)
Giving surgical mask to patient during examination	Never: 11 (9.17%)
	Sometimes: 18 (15.00%)
	Patients can use their own mask: 17 (14.17%)
	Only during colonoscopy: 11 (9.17%)
	Always: 63 (52.50%)
Negative pressure room	Yes: 1 (0.83%)
	No: 199 (99.17%)
Adequate ventilation	No: 6 (5.00%)
	Natural ventilation through opening windows: 90 (75.00%)
	Mixed mode of mechanical ventilation: 18 (15.00%)
	Air purification 6 (5.00%)
Cleaning and sterilization of air	Ultraviolet (UV) irradiation: 21 (17.50%)
	Ozone treatment: 2 (1.67%)

*COVID-19* coronavirus disease 19, *EUS* endoscopic ultrasound, *ERCP* endoscopic retrograde cholangiopancreatography, *ESGE* European Society of Gastrointestinal Endoscopy, *ESGENA *European Society of Gastroenterology and Endoscopy Nurses and Associats

**Fig. 1:**
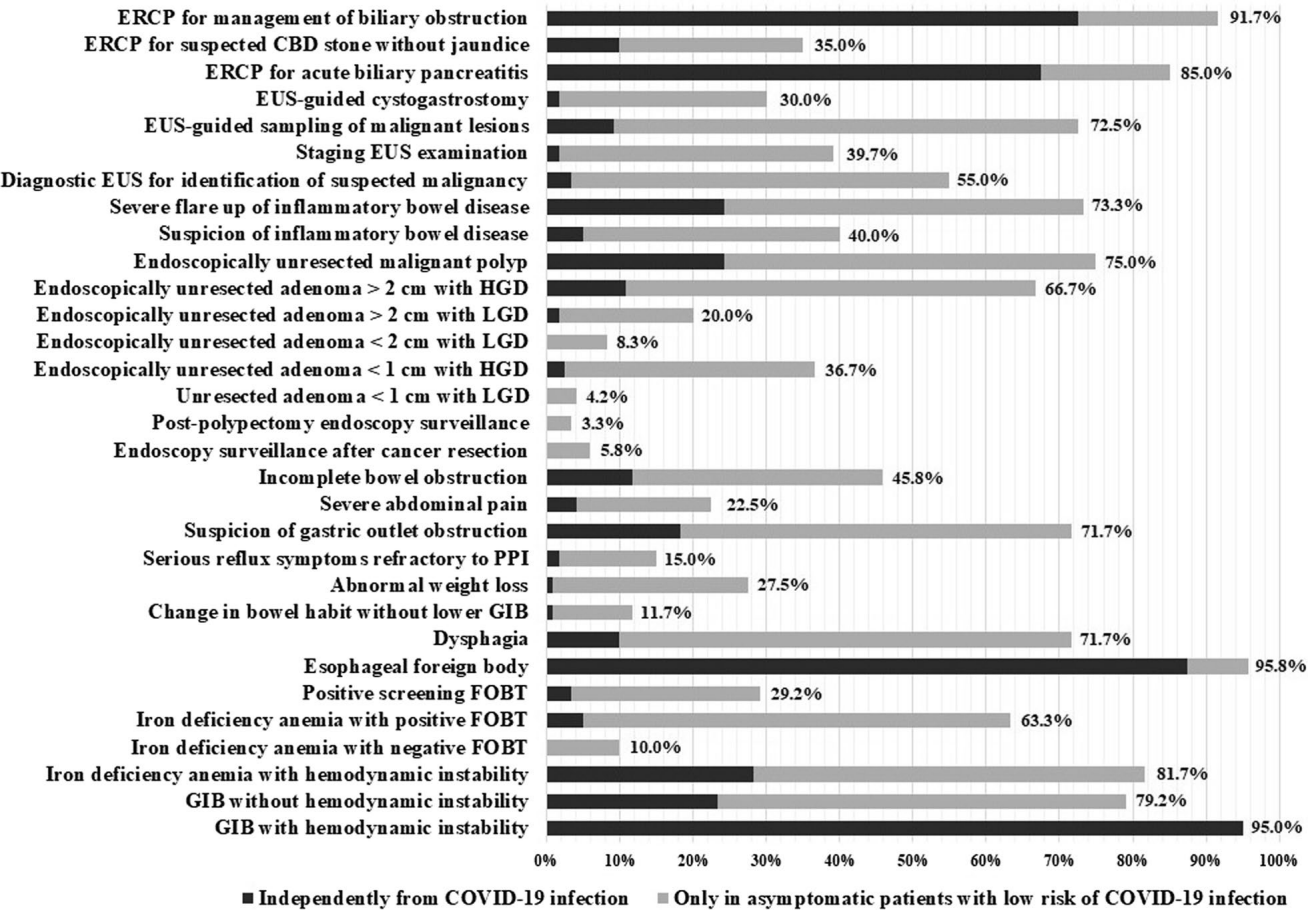
Indications for endoscopic procedures in which endoscopy cannot be postponed after the pandemic based on the opinion of Hungarian gastroenterologists. *ERCP* endoscopic retrograde cholangiopancreatography, *CBD* common bile duct, *EUS* endoscopic ultrasound, *HGD* high grade dysplasia, *LGD* low grade dysplasia, *PPI* proton pump inhibitor, *GIB* gastrointestinal bleeding, *FOBT* fecal occult blood test

Endoscopists considered that acute upper or lower gastrointestinal bleeding with hemodynamic instability (95.00%), foreign body in esophagus (92.50%), ERCP for the management of obstructive jaundice (91.67%) and biliary pancreatitis (85.00%) are the most important indications of endoscopy during COVID-19 pandemic. This correlates well with the position statement of ESGE and ESGENA. The other endoscopic indications were classified outstandingly important by less than one quarter of the answerers. The decision-making of endoscopists about the necessity of procedures is extremely influenced by the risk stratification of patients for possible COVID-19 infection. In cases of high-risk or positive patients, endoscopic examinations would be performed mostly if the above-mentioned main indications exist; however, agreement of endoscopists was weaker (gastrointestinal bleeding with hemodynamic instability 95.00%, foreign body in esophagus 87.50%, ERCP in obstructive jaundice 72.50%, ERCP in biliary pancreatitis 67.50%). Furthermore, about one quarter of the participants would also carry out the examination in cases of gastrointestinal bleeding without hemodynamic instability (23.33%), iron deficiency anemia with hemodynamic instability (28.33%), suspicion of gastric outlet obstruction (18.33%), endoscopically unresected malignant polyp (24.17%) and severe flare-up of inflammatory bowel disease (24.17%) (Fig. 1). The Hungarian population-based colorectal cancer screening program was launched in 2019. During COVID-19 pandemic, only 30% of endoscopic units continued the colonoscopic screening of patients with non-negative fecal occult blood test, but the performance of labs dropped below 50% compared to their previous capacity in 77.78% of cases.

### Quality of infection prevention and control strategies

**Fig. 2:**
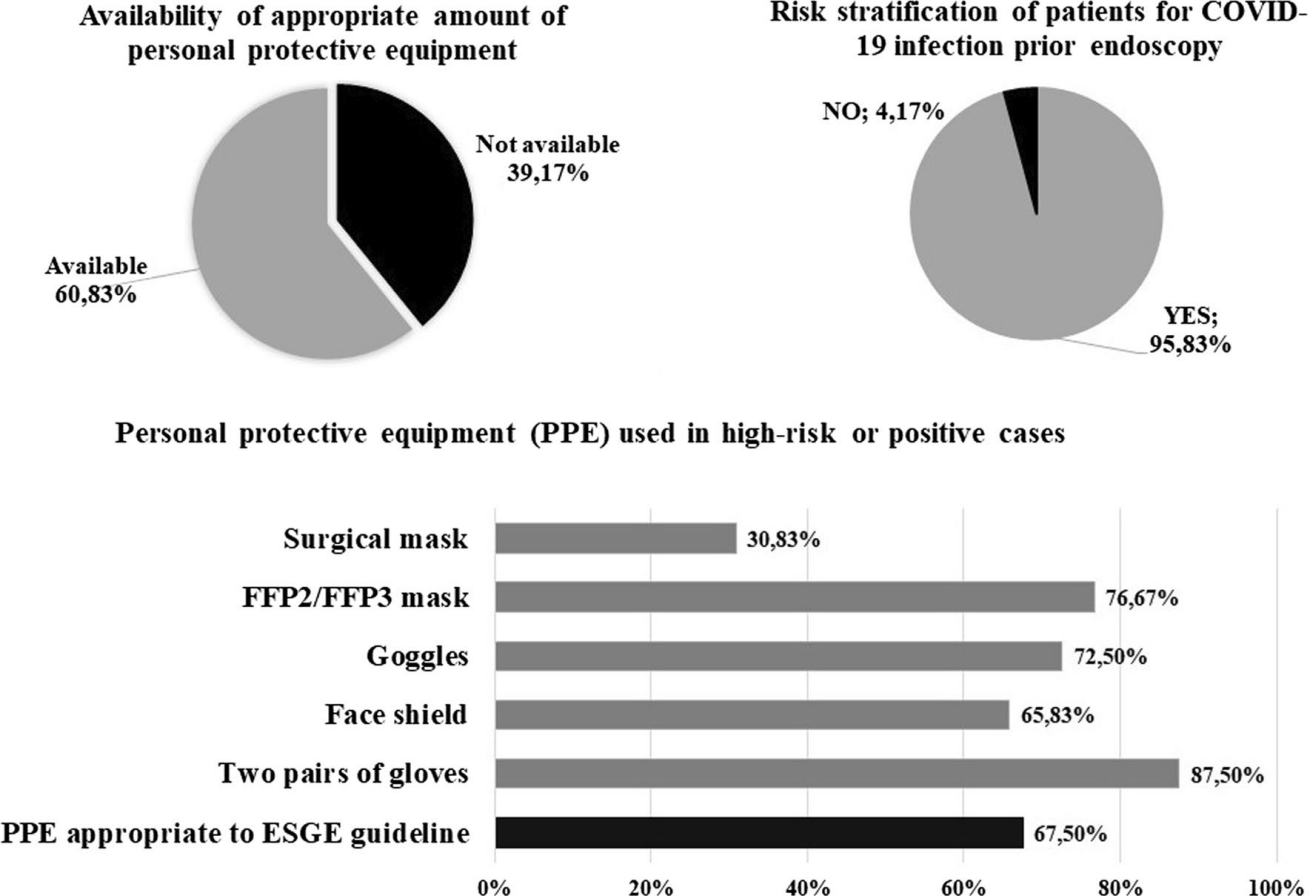
Availability and usage of personal protective equipment in Hungary based on the results of survey. *COVID-19* coronavirus disease 19, *ESGE* European Society of Gastrointestinal Endoscopy, *FFP* filtering facepiece

Total of 33.33% of the participants stated that they had undergone training about the prevention of COVID-19 infection in their workplace. The rate of institutes providing training was independent of the cumulative COVID-19 incidence of the region (low-, medium- and high risk regions: 34.88% vs. 17.86% vs. 42.86%, *p* = 0.117); in contrast, close correlation was observed with the capacity of labs (low-, medium- and high capacity labs: 21.88% vs. 35.29%; vs. 46.15%, *p* = 0.049). Overall 118 participants (98.33%) thought that staff of endoscopy units is at elevated risk of COVID-19 infection, however, there was no consensus about the risk of different endoscopic modalities: 55% of endoscopists said that the risk increase is the most pronounced in case of upper endoscopy (gastroscopy, ERCP, EUS), and in the rest of cases, they emphasized that the risk is similar in cases of upper and lower endoscopies and independent from the modality. 115 of the 120 participating gastroenterologists (95.83%) determined the risk of patients for COVID-19 infection one day prior to endoscopy in each cases, however in 14 cases (11.67%), they often had no opportunity to use risk-appropriate PPE. In 60.83% of answers, the availability of appropriate amount and quality of PPE in the endoscopy unit was reported, and it was not affected by cumulative incidence of COVID-19 infection of regions (low-, medium- and high risk regions: 63.27% vs. 57.14% vs. 60.47%, *p* = 0.868) and endoscopic capacity of labs (low-, medium- and high capacity labs: 61.70% vs. 41.18%; vs. 74.36%, *p* = 0.030). In cases of high-risk or positive patients, FFP-2/3 mask and protective eyewear were applied in 76.67% and 90.00%, respectively. The used PPE fully corresponded to the recommendation of ESGE and ESGENA in 67.50% of cases (Fig. 2). Negative-pressure room was available only in one institute. Ultraviolet irradiation and ozone treatment for the cleaning and sterilization of air and surfaces were used only in 17.50% and in 1.67% of endoscopy units. Based on the answers, the adequate ventilation and/or air purification was provided in 95% of cases which was made possible by natural ventilation through opening windows (75.00%), by mixed mode of mechanical ventilation (15.00%) or by air purification (5.00%).

## Discussion

Protection of health care professionals in endoscopy units has paramount importance during COVID-19 pandemic because endoscopies should be considered aerosol-generating procedures which facilitates the spreading of this respiratory pathogen virus. Several recommendations have been developed since the beginning of pandemic, which have been changing rapidly and differed from each other in several aspects. The national guidelines are substantially influenced by local regulations and rules about COVID-19 pandemic, as well the availability of human and material resources. Despite the wide availability of updated guidelines, the adherence of gastroenterologists to them are still questionable. The most important advantage of our survey study is that it anonymously examined the infection prevention and control strategies of endoscopic units in real-life setting based on the changes in endoscopic workflow, indications of examination, PPE, and disinfection techniques of labs.

Our results show that there is significant variability among gastroenterologists regarding indications requiring endoscopic procedures during COVID-19 pandemic and the application of PPE. The general strategy of recommendations is that only emergency endoscopies are permitted; however, the definition of urgency is often freely interpreted in the daily practice. According to the position statement of ESGE and ESGENA, endoscopy procedures should always be performed in cases of life-threatening disorders (e.g. gastrointestinal bleeding and anemia with hemodynamical instability, acute ascending cholangitis) and acute conditions such as foreign body in esophagus [5]. In addition, it determined a category of "high priority endoscopic procedures’ in which the examinations could be performed either immediately or postponed within 12 weeks based on case-by-case evaluation. This category is highly similar with the second indication category of the Hungarian recommendations (clinical conditions causing potentially permanent health damage if endoscopy is postponed), and the largest variability in the determination of timing of endoscopy could be observed in this group. An online survey carried out on social media platform (Twitter) well demonstrated this, where the answers of gastroenterologists about the timing of procedures showed an agreement of greater than 70% in only three of 16 scenarios [13]. Majority of respondents preferred the deferring of colonoscopy in cases of fecal occult blood test (FOBT) positivity and in cases of unresected colonic polyps. This complies with the guideline of British Society of Gastroenterology and American Gastroenterological Association [14, 15]. In contrast, ESGE recommends the endoscopic treatment of high-grade dysplasia (HGD) or early intramucosal cancer in the esophagus, stomach, or large colonic polyps at high-risk of submucosal invasion, as well the colonoscopy within organized CRC screening programs if the FOBT is positive. Our Hungarian survey found that more than 60% of endoscopists would perform the endoscopic treatment of malignant polyps and large colonic adenomas with HGD in cases of asymptomatic patients with low risk for COVID-19 infection. However, in high risk or positive patients, the indication of endoscopy is reduced to emergency situations. Low rate of respondents was still involved in the nationwide CRC screening program launched in 2019.

Compliance with the principle of “three zones and two passages” is generally recommended during the development of endoscopy unit and workflows. The contaminated zone, a potentially contaminated zone, and a clean zone should be clearly demarcated with adequate buffer areas in between [16]. COVID-19 is easily inactivated by commonly used disinfectants such as alcohol or chlorine-based solutions; therefore, no change in standard protocol is required in cleaning of endoscopes and reusable accessories [9, 17]. Full PPE includes disposable hair net, face shield/goggles, surgical masks or FFP-2/3 masks (depending on the patient's risk for infection), water-proof disposable gowns and gloves. In contrast, a survey carried out at the first weeks of pandemic in the United States highlighted that universal use of FFP-2/3 masks were used in only 65% of cases. Many endoscopists reported that use of FFP-2/3 masks was restricted to known or suspected COVID-19 cases due to limited availability (17%) or were not available at all (9%) [18]. Results of our survey carried out 6 weeks after the onset of COVID-19 pandemic in Hungary showed great similarity to the findings of this study. The appropriate amount and quality PPE were available in 60.83% of cases, and PPE used for examination of high-risk patients fully corresponded to the recommendation of ESGE in 67.50% of cases. The necessitate of PPE is also highlighted in the Iranian questionnaire survey. It involved approximately 480 gastroenterologist by March 26, 2020 and revealed that 10.6% of endoscopists had COVID-19 infections from which 60% had moderate disease, 30% had mild disease, and 10% had severe disease [19].

Our study has some major limitations which are in correlation with the anonymous, online nature of a survey study. The response rate to study invitation could not be exactly determined. All members of HSG received the invitation letter of the study because we have no detailed data about the gastroenterological profile of members (e.g. endoscopic assistant, basic scientist, practicing gastroenterologist, endoscopic experience and activity). The letter emphasized that enrollment of only endoscopists is planned in accordance with the aims of the investigation. The adequate representation of the whole country is not absolutely guarantied due to the relatively low number of participants; however, at least one gastroenterologist from each of the 19 counties of Hungary responded to the invitation, and the number of participants in the survey correlated with the number of endoscopy units.

## Conclusion

Our survey found large variability in indications of endoscopy and relative weak correspondence to national and international practical recommendations in terms of protective equipment. The endoscopic examination performed with inappropriate medical indication may also be risky for both patients and health care professionals. It should be emphasized that in high risk cases, about 10–20% of endoscopists would not carry out examinations which should not be postponed according to the recommendations of ESGE and HSG. It could increase the risk of patient's permanent health damage. In contrast, completion of postponable examinations of high risk or positive patients exposes the staff of endoscopy unit to unnecessary risk. In significant proportion of cases, the inadequate use of PPE is not related to lack of resources. Our findings suggest that adequate training about infection prevention could be beneficial, which can be further improved by the development of detailed guidance about the indications and timing of endoscopy adapted to local human and material resources and national pandemic regulations.

## Supplementary Information

**Additional file 1.** Questionnaire of the surveys-based multicentric nationwide study which evaluated the effect of COVID-19 pandemic on the endoscopic laboratory workflows and on the infection control of endoscopic units.

## Data Availability

The datasets used and/or analyzed during the current study are available from the corresponding author on reasonable request.

## Abbreviations

COVID-19: Coronavirus disease 2019
CRC: Colorectal cancer
EUS: Endoscopic ultrasound
ERCP: Endoscopic retrograde-cholangio-pancreatography
ESGE: European Society of Gastrointestinal Endoscopy
ESGENA: European Society of Gastroenterology and Endoscopy Nurses and Associates
FFP: Filtering facepiece
FOBT: Fecal occult blood test
HGD: High grade dysplasia
HSG: Hungarian Society of Gastroenterology
PPE: Personal protective equipments
