# Supplementary material for: Effect of COVID-19 pandemic on workflows and infection prevention strategies of endoscopy units in Hungary: a cross-sectional survey
Source: BMC Gastroenterol. 2021 Mar 3;21:98. doi: 10.1186/s12876-021-01670-3 (PMC7927759; doi:10.1186/s12876-021-01670-3)
Supplement: Supplementary file 1 — Additional file 1. Questionnaire of the surveys-based multicentric nationwide study which evaluated the effect of COVID-19 pandemic on the endoscopic laboratory workflows and on the infection control of endoscopic units. [file 12876_2021_1670_MOESM1_ESM.docx]

# Have you read the ESGE Position Statement on gastrointestinal endoscopy and the COVID-19 pandemic?

- 1. I did not know ESGE Position Statement
  2. No, I did not have time yet
  3. Yes, but I thought the same
  4. Yes, it was very helpful

# Does the COVID-19 pandemic affect the work in your endoscopic lab?

- 1. Yes
  2. No

# How many doctors working in your lab had to leave due to any reason related to COVID-19 pandemic (age > over 65, chr. disease)?

1. Nobody
2. < 20%
3. 21-40%
4. 41-60%
5. > 60%

# How many doctors worked originally in your endoscopic laboratory?

- 1. 1-3
  2. 4-6
  3. 7-10
  4. >10

1. How many assistants/technicians had to leave due to any reason related to COVID-19 pandemic (age > over 65)?
   1. Nobody
   2. < 20%
   3. 21-40%
   4. 41-60%
   5. > 60%

# How many assistants worked originally in your endoscopic laboratory?

- 1. 1-3
  2. 4-6
  3. 7-10
  4. >10

1. Does the reduced staff number affect your endoscopy unit’s workflow?
   1. Yes
   2. No
2. Do you think the staff of your endoscopic lab is at increased risk of COVID-19 infection?
   1. Yes
   2. No
3. In your opinion, which endoscopic procedure poses higher risk of infection for the staff?
   1. Upper GI endoscopy
   2. Lower GI endoscopy
   3. I am not sure
   4. Both pose the same risk
4. In your opinion, which endoscopic procedure poses the highest risk of infection for the staff?
   1. Gastroscopy
   2. ERCP
   3. Colonoscopy
   4. Endoscopic ultrasound
   5. The risk is the same
   6. I am not sure
5. Did you attend or participate in any advanced training at your workplace on the management of the endoscopic lab during the pandemic?
   1. Yes
   2. No
6. How many endoscopic examinations did you perform in the last 1 week?
   1. 0
   2. 1-5
   3. 6-10
   4. 11-15
   5. >15
7. What is the percentage decrease compared to your previous endoscopic capacity?
   1. < 25%
   2. 25-50%
   3. 51-75%
   4. >75%
8. What type of endoscopic examination decreased the most in your practice?
   1. All of them
   2. Colonoscopy
   3. Gastroscopy
   4. ERCP
   5. Endoscopic ultrasound
9. Do you perform a risk stratification for COVID infection before endoscopy?
   1. Yes
   2. No
10. If yes, does the result of the risk stratification affect what protective steps you take during the examinations?
    1. No, I am not afraid of infection
    2. No, because I do not have proper protective equipment anyway
    3. Yes, I change the mask
    4. Yes, I change the protective clothing
11. Does your lab have enough protective equipment?
    1. Not at all
    2. No, although we tried to get it on our own
    3. Yes, because everything is provided centrally
    4. Yes, because we obtained everything
12. Are there less people in the endoscopic room during the examinations since the outbreak of COVID-19 pandemic?
    1. Yes, only the endoscopist and the required number of assistants are in
    2. No, the administrator is still in
    3. No, the trainee is still in
    4. No, the patients’ relatives can be inside
13. During an endoscopic examination, how many people are usually in the room except for the patient?
    1. Two
    2. It depends on the type of the examination, two or three
    3. Four
    4. Even more
14. Does the patient wear a surgical mask in the endoscopic lab?
    1. No
    2. Yes, if he/she brought one
    3. Sometimes we give them
    4. Only during colonoscopy
    5. Always, also in case of gastroscopy, before and after the examination
15. Is there a negative-pressure room in your endoscopic lab?
    1. Yes
    2. No
16. If not, is adequate ventilation provided in the endoscopic lab?
    1. Yes, with opened windows
    2. Yes, with ventilator on the outside
    3. Yes, with air filter
    4. Uninsured
17. Is decontamination (eg UV-C, Ozone, etc.) used in the room to prevent aerosol infection?
    1. Why, should it be?
    2. Ozone
    3. UV-C
    4. Other
    5. We do not use anything
18. If the answer to the previous question is other, which disinfectant option is used to prevent aerosol infection?

____________________________________________________________________________________________________________________________________________________________________________________________________________________________________________________________________________________

1. What do you think is the most important information and symptom to consider a patient to be at high risk?
   1. Travel abroad within a month
   2. Prolonged fever
   3. Cough
   4. Diarrhea
   5. COVID suspicious or positive close contact
2. What do you wear during the examination of a high-risk patient?
   1. I use a surgical mask
   2. I use an FFP 2-3 mask
   3. Eye protector glasses
   4. I use face-shield
   5. I use double gloves
3. Have you ever tested a confirmed COVID-19 patient?
   1. Yes
   2. No
4. If yes, have you changed your protective equipment compared to the examination of a high-risk patient?
   1. Yes
   2. No
5. If yes, how did you change your protective equipment:

________________________________________________________________________________________________________________________________________________________________________________________________________________________

1. Is there a colleague or nurse in your lab who has already been SARS-CoV-2 positive?
   1. No
   2. Yes, occurred
   3. Yes, I know more cases
2. In your opinion, which of the following indications cannot be postponed after the pandemic?
   1. Lower / upper GI bleeding with hemodynamic instability
   2. Lower / upper GI bleeding without hemodynamic instability
   3. Iron deficiency anemia with hemodynamic instability
   4. Iron deficiency anemia without hemodynamic instability, gFOBT/FIT negative
   5. Iron deficiency anemia, gFOBT/FIT positive
   6. gFOBT/FIT positive, screening program
   7. Foreign body in esophagus
   8. Dysphagia
   9. Change in bowel habit without bloody stools
   10. Weight loss
   11. Symptoms of severe GERD
   12. Suspicion of gastroparesis
   13. Severe abdominal pain
   14. Subileus
   15. CRC postoperative control
   16. Post-polypectomy surveillance
   17. Endoscopically confirmed adenoma, ≤1 cm in size, low-grade dysplasia
   18. Endoscopically confirmed adenoma, ≤1 cm in size, high-grade dysplasia
   19. Endoscopically confirmed adenoma, ≤2 cm in size, low-grade dysplasia
   20. Endoscopically confirmed adenoma, >2 cm in size, low-grade dysplasia
   21. Endoscopically confirmed adenoma, >2 cm in size, high-grade dysplasia
   22. Endoscopically confirmed malignant adenoma
   23. Suspected inflammatory bowel disease
   24. Severe exacerbation of inflammatory bowel disease, refractory to treatment
   25. Diagnostic EUS in suspected malignancy
   26. EUS staging for known malignancy
   27. EUS sampling in case of suspected tumor
   28. EUS-guided cyst drainage
   29. ERCP in acute biliary pancreatitis
   30. ERCP for choledocholithiasis without jaundice
   31. ERCP in obstructive jaundice
3. In your opinion which is the first 5 most important indications of endoscopy?
   1. Lower / upper GI bleeding with hemodynamic instability
   2. Lower / upper GI bleeding without hemodynamic instability
   3. Iron deficiency anemia with hemodynamic instability
   4. Iron deficiency anemia without hemodynamic instability, gFOBT/FIT negative
   5. Iron deficiency anemia, gFOBT/FIT positive
   6. gFOBT/FIT positive, screening program
   7. Foreign body in esophagus
   8. Dysphagia
   9. Change in bowel habit without bloody stools
   10. Weight loss
   11. Symptoms of severe GERD
   12. Suspicion of gastroparesis
   13. Severe abdominal pain
   14. Subileus
   15. CRC postoperative control
   16. Post-polypectomy surveillance
   17. Endoscopically confirmed adenoma, ≤1 cm in size, low-grade dysplasia
   18. Endoscopically confirmed adenoma, ≤1 cm in size, high-grade dysplasia
   19. Endoscopically confirmed adenoma, ≤2 cm in size, low-grade dysplasia
   20. Endoscopically confirmed adenoma, >2 cm in size, low-grade dysplasia
   21. Endoscopically confirmed adenoma, >2 cm in size, high-grade dysplasia
   22. Endoscopically confirmed malignant adenoma
   23. Suspected inflammatory bowel disease
   24. Severe exacerbation of inflammatory bowel disease, refractory to treatment
   25. Diagnostic EUS in suspected malignancy
   26. EUS staging for known malignancy
   27. EUS sampling in case of suspected tumor
   28. EUS-guided cyst drainage
   29. ERCP in acute biliary pancreatitis
   30. ERCP for choledocholithiasis without jaundice
   31. ERCP in obstructive jaundice
4. In your opinion, which endoscopic examination should be performed in a confirmed, SARS-CoV-2 positive patient?
   1. Lower / upper GI bleeding with hemodynamic instability
   2. Lower / upper GI bleeding without hemodynamic instability
   3. Iron deficiency anemia with hemodynamic instability
   4. Iron deficiency anemia without hemodynamic instability, gFOBT/FIT negative
   5. Iron deficiency anemia, gFOBT/FIT positive
   6. gFOBT/FIT positive, screening program
   7. Foreign body in esophagus
   8. Dysphagia
   9. Change in bowel habit without bloody stools
   10. Weight loss
   11. Symptoms of severe GERD
   12. Suspicion of gastroparesis
   13. Severe abdominal pain
   14. Subileus
   15. CRC postoperative control
   16. Post-polypectomy surveillance
   17. Endoscopically confirmed adenoma, ≤1 cm in size, low-grade dysplasia
   18. Endoscopically confirmed adenoma, ≤1 cm in size, high-grade dysplasia
   19. Endoscopically confirmed adenoma, ≤2 cm in size, low-grade dysplasia
   20. Endoscopically confirmed adenoma, >2 cm in size, low-grade dysplasia
   21. Endoscopically confirmed adenoma, >2 cm in size, high-grade dysplasia
   22. Endoscopically confirmed malignant adenoma
   23. Suspected inflammatory bowel disease
   24. Severe exacerbation of inflammatory bowel disease, refractory to treatment
   25. Diagnostic EUS in suspected malignancy
   26. EUS staging for known malignancy
   27. EUS sampling in case of suspected tumor
   28. EUS-guided cyst drainage
   29. ERCP in acute biliary pancreatitis
   30. ERCP for choledocholithiasis without jaundice
   31. ERCP in obstructive jaundice
5. Did your endoscopic unit continue colonoscopies in patient with non-negative FIT test in the organized national CRC screening program?
   1. Yes
   2. No
6. If yes, what is the percentage decrease compared to your previous capacity?
   1. <25%
   2. 25-50%
   3. 51-75%
   4. > 75%
7. Do you work in a private endoscopic lab as well?
   1. Yes
   2. No
8. If yes, did you perform endoscopies there at the last week?
   1. Yes
   2. No
9. In your opinion, does a decrease in the number of endoscopic examinations and the omission of certain examinations pose a significant risk to patients?
   1. No, so far we have performed too many endoscopies with too liberal indications
   2. No, those few months don't matter
   3. If we choose the indications well, then not necessarily
   4. Yes, certainly there will be some patients whose delay in the diagnosis causes harm to their health
   5. Yes, due to the pandemic, many patients will not be examined in an adequate time, so we will face many advanced tumors
10. In your opinion, what will be the expected workload in the endoscopic laboratories 6 months after the end of the COVID-19 pandemic?
    1. A pre-epidemic load is expected
    2. Even after the pandemic, many people will be scared, which will result in fewer patients at first, than before
    3. Significant growth is expected right away, but we will be able to handle it
    4. The capacity of the lab will not be able to withstand the load
11. How long do you think the COVID-19 pandemic will last?
    1. 1 month
    2. 3 months
    3. 6 months
    4. 12 months
